# Introducing professional drug information resources to non-healthcare undergraduates: a case report on promoting drug information literacy

**DOI:** 10.5195/jmla.2026.2165

**Published:** 2026-01-01

**Authors:** Hey Young Rhee, Kiyon Rhew

**Affiliations:** 1 jonju@dongduk.ac.kr, College of Social Sciences Div. of Library & Information Science, Dongduk Women's University, Republic of Korea; 2 kiyon@dongduk.ac.kr, College of Pharmacy, Professor, College of Pharmacy, Dongduk Women's University, Republic of Korea

**Keywords:** Drug information database, non-healthcare students, health literacy, Evidence based practice, Health professionals

## Abstract

**Background:**

Non-healthcare undergraduate students frequently seek drug-related information online, often relying on unverified sources such as Google or YouTube. Early exposure to professional drug information databases may promote evidence-based information-seeking habits.

**Case Presentation:**

A one-hour training session on using Lexicomp, a professional drug information database, was conducted for 55 non-healthcare students and 58 pharmacy students at a women's university in South Korea. The session included live demonstrations and guided search tasks. Participants completed pre- and post-training surveys assessing their information-seeking behaviors, perceptions of source reliability, and intention to use Lexicomp. Students also ranked drug information types they typically searched for and anticipated using Lexicomp to find. Only 1.8% of non-healthcare students had prior knowledge of Lexicomp, compared to 100% of pharmacy students. After the training, 100% of non-healthcare students rated Lexicomp as more reliable than their usual sources, and over 90% expressed willingness to use it in the future. A marked shift in information-seeking priorities was observed, with greater emphasis on clinically relevant topics such as adverse effects and contraindications. Students reported increased confidence and found the platform easier to use than expected.

**Conclusion:**

A brief educational intervention was effective in improving drug information literacy among non-healthcare students. Early training in professional resources may foster long-term adoption of evidence-based practices in personal health information use.

## BACKGROUND

In today's digital environment, undergraduate students frequently seek health-related information through online platforms. While this increased accessibility can empower individuals to make informed health decisions, it also exposes them to significant risks associated with misinformation, particularly from unverified sources such as general search engines, social media, and generative AI tools [1,2]. Generative AI, in particular, has rapidly emerged as a popular tool for retrieving quick answers to health queries [3,4]. However, studies show that the reliability and accuracy of AI-generated health summaries remain inconsistent and potentially misleading [5,6]. In addition to generative AI tools, students frequently rely on general search engines (e.g., Google), video-sharing platforms (e.g., YouTube), and regional web portals (e.g., Naver) for drug-related information; however, these sources are also prone to misinformation and variable quality [7,8]

A growing body of research suggests that individuals tend to continue using the first information source they encounter, a phenomenon influenced by cognitive biases such as anchoring and source loyalty [9,10]. Over time, users may become accustomed to a particular level or quality of information, even if that source lacks scientific credibility [11]. This pattern is especially concerning among university students, who are at a formative stage in developing lifelong habits around information seeking and evaluation.

This concern is particularly acute when it comes to drug-related information, where inaccurate details about dosage, interactions, or contraindications may directly impact patient safety. Currently, most professional drug information databases, such as Lexicomp, are primarily used by healthcare professionals and students in related disciplines. However, at many institutions, non-healthcare students remain unaware of these resources despite having access through university subscriptions. Educating undergraduates, especially those outside healthcare disciplines, on how to navigate professional databases can enhance their ability to evaluate drug-related information critically and make more evidence-based health decisions during and beyond their academic years.

This case report describes an educational intervention conducted at a South Korean university, where a pharmacy program is the only health professional major. The goal was to assess whether a brief training in the use of Lexicomp could improve non-healthcare students' awareness, attitudes, and future intent to use professional drug information resources.

## CASE PRESENTATION

This case report describes a single-session educational intervention designed to improve drug information literacy among undergraduate students, particularly those without a healthcare background. The intervention was implemented at a private women's university in South Korea, where the College of Pharmacy is the only health-related academic program. Other departments include disciplines such as humanities, social sciences, business, and natural sciences. As the university does not have medical or nursing schools, the study population consisted exclusively of pharmacy majors and students from non-healthcare departments.

A total of 113 undergraduate students voluntarily participated in the study. Of these, 58 were pharmacy majors, while 55 were non-healthcare majors. Most students were in their second to fourth year of study. Prior to the intervention, all participants had institutional access to Lexicomp Online, a widely used subscription-based professional drug information database, through the university library. However, nearly all non-healthcare students were unaware of the database or its potential use in verifying medication-related information.

The intervention consisted of a 60-minute in-person training session delivered by a faculty member specializing in clinical pharmacy. The session was conducted in a classroom setting equipped with a projector and internet access. The educational content was carefully tailored to introduce the concept of reliable, evidence-based drug information, to contrast it with unverified sources often used by the general public, and to provide practical instruction on how to navigate the Lexicomp platform.

The session began with a brief lecture highlighting common issues associated with relying on unverified or incomplete drug information. This included examples of misinformation from general websites and the potential risks of such reliance, particularly in the context of patient self-medication. This was followed by a live demonstration of the Lexicomp interface, during which the instructor showed how to search for a drug and locate specific types of information. Key sections introduced during the demonstration included drug indications, contraindications, dosage and administration, adverse effects, drug-drug and drug-food interactions, warnings and precautions, and patient education leaflets about medication or disease.

After the demonstration, participants were guided through a set of practice exercises using their own devices or shared screens. They were asked to search for information on commonly used medications, such as ibuprofen or loratadine, and to locate specific content such as appropriate dosing for different age groups, potential interactions with alcohol, or patient counseling points. The instructor provided real-time feedback and clarification as needed. This hands-on component was designed to reinforce the navigation and interpretive skills necessary to retrieve accurate drug information independently.

Although both pharmacy and non-healthcare students received the same training content, the focus of the intervention for non-healthcare students was to raise awareness and promote confidence in using professional-level drug information tools. The aim was not to train them as healthcare providers. Instead, the goal was to support safe and informed decision-making as health information consumers.

To evaluate the effects of the intervention, all participants completed an anonymous pre-training survey that assessed their prior awareness of Lexicomp, their usual sources of drug information, and their perceptions of reliability and usefulness. A post-training survey was completed within two weeks of the session, including parallel questions and additional items on satisfaction, future use intentions for Lexicomp, and the perceived value of resource access for non-healthcare students. The survey items used 5-point Likert scales, ranging from 1 (strongly disagree) to 5 (strongly agree), and included internal consistency checks. Although long-term outcomes such as retention of information or behavior change were not evaluated, the intervention aimed to assess the short-term shift in perceptions and attitudes following structured exposure to a professional database.

This study was conducted in accordance with the principles of the Declaration of Helsinki and was approved by the Dongduk Women's University's institutional review board (IRB No. DDWU2403-01).

## EVALUATION AND OUTCOMES

To evaluate the effectiveness of the educational intervention, participants completed a structured survey both before and after the training session. The pre-training survey assessed their prior experience with and awareness of professional drug information sources, typical information-seeking behaviors, perceived reliability of commonly used sources (e.g., Google, YouTube, Naver, generative AI), and general interest in drug-related topics. The post-training survey repeated several of the same items and added questions regarding satisfaction with the training, perceived ease of use of Lexicomp, and future intention to use the database (Table 1).

**Table 1:** The results of pre-training survey on the use of a drug information database

Questions		Non healthcare major students (n=55)	Pharmacy students (n=58)	p-value
How interested are you in health, medicine, and diseases in general?		3.8727 (0.7467)	4.1379 (0.9262)	0.0977
Compared to your peers, how much more interested are you in health, medicine, and diseases?		3.6909 (0.8579)	4.2241 (0.7503)	0.0006
Have you ever searched for information about your own health, the health of those around you, or medications they are taking?	No	0 (0)	0 (0)	N/A
	Yes	55 (100)	58 (100)	
If yes for above question, how frequently do you search for such information?	≥ once a week	9 (16.36)	6 (10.34)	0.0561
	≥ once monthly, < once a week	13 (23.64)	18 (31.03)	
	≥ once quarterly, < once monthly	31 (56.36)	24 (41.38)	
	< once quarterly	2 (3.64)	10 (17.24)	
What platforms do you use for information searches?	General portal websites such as Naver or Google	49 (89.09)	36 (62.07)	0.0005
	Media-sharing platforms such as YouTube	5 (9.09)	6 (10.34)	
	Professional drug information resources	1 (1.82)	16 (27.59)	
What sources do you use for information searches?	Personal channels run by healthcare professionals	29	17	0.0796
	Private organizations such as drug information centers	11	17	
	Government agencies such as the MFDS*	9	12	
	Specialized drug information database	6	12	
How satisfied are you with the sources of information you use?		3.6909 (0.6047)	3.7069 (0.4592)	0.8750
How much do you trust the health or drug-related information from your sources?		3.8182 (0.5474)	3.7931 (0.4086)	0.7840
Were you aware that you could use a professional drug information database through our school library?	No	54 (98.18)	6 (10.34)	<.0001
	Yes	1 (1.82)	52 (89.66)	
Have you ever felt the need for a more professional or reliable source of information when searching for health or medication-related information?	No	4 (7.27)	6 (10.34)	0.5655
	Yes	51 (92.73)	52 (89.66)	

Among non-healthcare majors, 1 out of 55 students (1.8%) reported having heard of professional drug information database such as Lexicomp prior to the training, and none had used it. In contrast, all 58 pharmacy students were already familiar with the database and had used it at least once for coursework or personal study. After the session, 100% (55 students) of non-healthcare students reported that Lexicomp was more reliable than the sources they previously used, and over 90% (50 students) expressed a willingness to use it in the future, especially when seeking information about drug side effects, dosage, or interactions.

The post-training responses indicated a marked shift in attitudes among non-healthcare students. The average perceived reliability of Lexicomp was rated significantly higher than that of general internet sources, with a mean score of 4.49 out of 5 (standard deviation (SD) 0.61). In addition, non-healthcare students reported feeling more confident in their ability to locate and interpret drug-related information using a professional interface. When asked whether they believed it was valuable for students outside of healthcare fields to have access to such databases, the average Likert score was 4.15, indicating a high level of perceived relevance and benefit (Table 2).

**Table 2:** The results of post-training survey on the use of a drug information database

Questions		Non healthcare major students (n=55)	Pharmacy students (n=58)	p-value
How interested are you in health, medicine, and diseases in general?		3.9091 (0.8449)	4.2586 (0.6898)	0.0174
Compared to your peers, how much more interested are you in health, medicine, and diseases?		3.8182 (0.9830)	4.1586 (0.8231)	0.04908
How satisfied are you with the training on the professional drug information database?		4.5091 (0.5733)	4.9138 (0.2831)	<.0001
How satisfied are you with the professional drug information database itself?		4.3273 (0.6953)	4.8103 (0.3955)	<.0001
Do you think the professional drug information database is more reliable than the sources you previously used?	No	0 (0)	0 (0)	N/A
	Yes	55 (100)	58 (100)	
Do you expect to use the professional drug information database, or have you already used it?	No	5 (9.09)	5 (8.62)	0.9299
	Yes	50 (90.91)	53 (91.38)	
Do you think the professional drug information database should be provided to undergraduate students outside of healthcare-related fields?		4.1455 (0.5584)	4.7759 (0.4207)	<.0001
Do you think there is a possibility that undergraduate students outside healthcare-related fields will use the professional drug information database?		3.8545 (0.9112)	4.5000 (0.5044)	<.0001
How frequently do you expect to use the professional drug information database?	≥ once a week	2 (3.64)	22 (37.93)	<.0001
	≥ once monthly, < once a week	25 (45.45)	24 (41.38)	
	≥ once quarterly, < once monthly	19 (34.55)	12 (20.69	
	< once quarterly	9 (16.36)	0 (0)	
Do you plan to inform your peers about the existence of the professional drug information database provided by the university library?	No	1 (1.82)	0 (0)	0.3023
	Yes	54 (98.18)	58 (100)	
Do you plan to recommend the use of the professional drug information database to your peers?	No	0 (0)	0 (0)	N/A
	Yes	55 (100)	58 (100)	

This attitudinal shift was also reflected in students’ prioritization of drug-related information types. As shown in Figure 1A, prior to the intervention, students most frequently searched for drug efficacy, followed by adverse effects and dosage. Before the intervention, students mainly relied on general platforms such as Google or YouTube, where information can be incomplete or inaccurate and sometimes provided by non-experts or community sources. After the training, as depicted in Figure 1B, however, they reported a stronger intention to search for clinically critical topics such as adverse effects, contraindications, and drug interactions using professional drug information resources.

While long-term outcomes were not directly assessed, this shift suggests the potential for students to develop stronger skills in evaluating information and may encourage more evidence-based approaches to drug information seeking in the future. Notably, the expectation to use Lexicomp for wellness or health information also emerged among non-healthcare students, suggesting a broader understanding of the database's scope. This comparison between actual past behavior and intended future use underscores the potential of even a short instructional intervention to recalibrate students’ information-seeking behaviors toward more structured and evidence-based resources.

Satisfaction with the training session was also high. The overall satisfaction score for non-healthcare students was 4.51 out of 5 (SD 0.57), and qualitative feedback noted that the Lexicomp interface was easier to use than expected. Many participants appreciated the clarity and structure of the information provided and expressed surprise at the level of detail available in patient education materials.

Pharmacy students, who were included primarily as a reference group, showed little change between pre- and post-surveys, which was expected given their prior exposure to Lexicomp. Their survey data, however, provided a useful benchmark for interpreting the responses from non-healthcare students and highlighted the potential for convergence in information-seeking patterns when non-healthcare students are appropriately trained.

Reliability testing of the survey instrument showed acceptable internal consistency, with Cronbach's alpha calculated at 0.84 for repeated items assessing health interest and trust in information sources. To analyze changes in survey responses, descriptive statistics were used to summarize distributions, and independent t-tests were applied to compare post-training differences between healthcare and non-healthcare students. This suggests that the survey responses were stable and reflective of participants’ attitudes.

Overall, the brief, single-session intervention resulted in substantial improvements in awareness, perceived usefulness, and intended use of Lexicomp among non-healthcare students, suggesting that even limited exposure can positively impact drug information literacy when supported by institutional access and guided instruction.

## DISCUSSION

This case illustrates the feasibility and impact of providing structured training on a professional drug information database to undergraduate students without a healthcare background. The intervention demonstrated that even a single, brief instructional session can significantly improve non-healthcare students’ awareness of high-quality drug information sources, their trust in those sources, and their willingness to use them in future information-seeking tasks. These findings support the idea that professional resources like Lexicomp, though originally developed for clinical use, can also be valuable tools for improving drug information literacy in the general student population when accompanied by guided instruction [12–14].

The results further highlight the underutilization of institutionally licensed databases by students outside of the health sciences, despite their availability. Prior to training, nearly all non-healthcare participants relied on general search engines or social media platforms to obtain drug-related information. This pattern reflects broader trends in consumer health information-seeking behavior, where convenience and familiarity often outweigh concerns about accuracy or source credibility. As noted in prior cognitive science and information behavior research, individuals tend to stick with information sources they have used before, a tendency reinforced by anchoring effects, source loyalty, and cognitive effort minimization [9,10].

Introducing high-quality, structured databases like Lexicomp early in students’ academic experience may help to counteract such patterns by establishing higher standards for what constitutes credible information. When students are given the opportunity to interact with professional-level tools in an accessible, low-stakes environment, they are more likely to incorporate these resources into their regular information-seeking behaviors [15,16]. This may have broader implications for public health, as young adults increasingly manage aspects of their health independently and make decisions regarding self-medication, over-the-counter drug use, and interpreting medical advice found online [17,18].

Importantly, this intervention was not intended to train non-healthcare students as clinicians, nor to promote Lexicomp as a consumer resource. Rather, the goal was to support informed and safe health-related decisions by improving students’ ability to recognize, access, and evaluate professional drug information. The overwhelmingly positive reception from participants, along with the measurable increase in awareness and trust, suggests that similar interventions could be implemented at other institutions with minimal cost and high potential impact.

Several limitations must be acknowledged. The study involved a single institution with a relatively small and homogenous sample (i.e., students from a women's university in South Korea). The evaluation focused on short-term perceptual changes immediately following the intervention, and no follow-up was conducted to assess retention, continued use, or changes in actual behavior. Additionally, the study used self-reported measures, which may be subject to response bias. Future research could benefit from longer-term tracking of student behavior and comparisons across institutions or educational formats (e.g., online vs. in-person training). Nonetheless, the findings underscore the value of integrating drug information literacy training into general education curricula, particularly at universities with access to high-quality resources. Expanding these efforts may help bridge the information gap between healthcare professionals and the general public, reduce reliance on unreliable sources, and promote more evidence-based decision-making in everyday life.

This case highlights the value of introducing professional drug information databases, such as Lexicomp, to undergraduate students early in their academic journey. Even a brief, structured training session helped students, particularly those without a healthcare background, develop greater awareness of reliable sources, improved trust in evidence-based content, and a stronger willingness to use professional tools when seeking drug-related information. Equipping students with the ability to navigate clinically grounded resources may shape their long-term information-seeking behaviors, encouraging them to make health decisions based on credible evidence rather than unverified online content. As students increasingly manage aspects of their own health and support others in doing so, early education in drug information literacy can serve as a foundation for safer, more informed use of health information in the future.

## ETHICS STATEMENT

This study was conducted in accordance with the principles of the Declaration of Helsinki and was approved by the Dongduk Women's University's institutional review board (IRB No. DDWU2403-01). Written informed consent was obtained from all participants prior to their involvement.

## Data Availability

The data supporting the findings of this study are not publicly available due to privacy considerations but are available from the corresponding author on reasonable request.
